# Ibrutinib inhibits the replication of multiple poxviruses by targeting the Bruton tyrosine kinase

**DOI:** 10.1128/jvi.00517-25

**Published:** 2025-08-20

**Authors:** Kang Niu, Xiru Wang, Qiwei Jiang, Kai Liu, Shijie Xie, Baifen Song, Wenxue Wu, Xiao Li, Chen Peng

**Affiliations:** 1 National Key Laboratory of Veterinary Public Health and Safety, College of Veterinary Medicine, China Agricultural University 630101 , Beijing, China; 2 State Key Laboratory of Pathogen and Biosecurity, Key Laboratory of Jilin Province for Zoonosis Prevention and Control, Changchun Veterinary Research Institute, Chinese Academy of Agricultural Sciences 595703 , Changchun, China; Northwestern University Feinberg School of Medicine, Chicago, Illinois, USA

**Keywords:** Ibrutinib, BTK, mpox virus, lumpy skin disease virus, antiviral action

## Abstract

The recent surge in global monkeypox (mpox) outbreaks highlights the critical need for developing antiviral agents targeting orthopoxvirus infections. Ibrutinib, a selective Bruton tyrosine kinase (BTK) inhibitor initially approved by the FDA in 2013 for treating chronic lymphocytic leukemia (CLL) and mantle cell lymphoma (MCL), has emerged as a potential antiviral candidate based on a high-throughput screening for its efficacy against vaccinia virus (VACV). This study evaluated the antiviral capability of ibrutinib against VACV, mpox virus (MPXV), and lumpy skin disease virus (LSDV), demonstrating its strong antiviral activity against multiple poxviruses. *In vitro* assays confirmed that ibrutinib significantly inhibited the replication of VACV, MPXV, and LSDV across multiple cell lines. *In vivo* studies using VACV-infected BALB/c mice revealed that ibrutinib treatment extended survival, mitigated weight loss and lesion formation, and reduced viral loads in infected mice. Mechanistic investigations indicated that ibrutinib inhibited viral early and late protein synthesis as well as DNA replication. Furthermore, BTK manipulation influenced viral replication, underscoring ibrutinib’s antiviral action through a cellular target. Notably, BTK phosphorylation and nuclear translocation were observed during poxvirus infection, suggesting its role in viral replication. Overall, our findings suggest that ibrutinib is a promising candidate for treating poxvirus infections and that its cellular target, BTK, contributes to poxvirus replication.

**IMPORTANCE:**

Poxviruses are important zoonotic pathogens affecting human and animal health. The recent outbreak of monkeypox virus (MPXV) highlights the persistent threat posed by poxviruses to public health. In addition, lumpy skin disease virus (LSDV) causes economic impact by affecting the cattle industry. Currently, there are no effective drugs to combat LSD, which poses a major challenge to livestock health and productivity. Therefore, there is an urgent need to develop antiviral drugs against multiple poxviruses. Through high-throughput screening of antiviral drugs targeting vaccinia virus (VACV), we identified ibrutinib as a candidate antiviral drug. In our study, ibrutinib effectively inhibited the replication of MPXV, VACV, and LSDV *in vitro* and *in vivo*. Knockdown of Bruton tyrosine kinase (BTK), the cellular target of ibrutinib, significantly inhibited virus replication, while overexpression of BTK enhanced virus replication, which displays its role as a pro-viral effector. Overall, ibrutinib is a promising anti-poxvirus agent that can combat various poxviruses by targeting BTK.

## INTRODUCTION

Poxviruses are significant zoonotic pathogens that impact both human and animal health. Despite the eradication of smallpox in the 1980s (1), the recent outbreaks of mpox virus (MPXV) underscore the persistent threat posed by poxviruses to public health. The poxviridae family, as defined by the 2021 International Committee on Taxonomy of Viruses (ICTV), encompasses 22 genera and 83 species (2). The orthopoxvirus genus includes 12 recognized species, with notorious human pathogens such as variola virus, the causative agent of smallpox, and MPXV, which has led to regular endemic outbreaks in sub-Saharan Africa and global outbreaks since 2022.

Smallpox historically caused the most fatalities among infectious diseases, claiming approximately 300 million lives in the first 80 years of the 20th century (3). Although smallpox was declared eradicated in 1980, the discontinuation of smallpox vaccination has increased individuals’ vulnerability to other orthopoxviruses due to the loss of cross-protection (4). As demonstrated by the ongoing global mpox outbreak, orthopoxviruses still present a public health risk, with more than 87,000 cases reported in over 100 countries. Apart from orthopoxviruses, other poxviruses, such as lumpy skin disease virus (LSDV), cause economic impact by primarily affecting the cattle industry (5–9). Currently, there are no effective drugs to combat LSD, posing a significant challenge to livestock health and productivity.

Bruton tyrosine kinase (BTK) is a non-receptor tyrosine kinase belonging to the TEC family of kinases, highly conserved across evolution (10). BTK expression is notably elevated in malignant B cells, playing a critical role not only in the differentiation and proliferation of B cells but also in inhibiting Fas/CD95-induced apoptosis of B lymphocytes (11). This insight has led to the development of BTK inhibitors (BTKi) for the treatment of B-cell malignancies. Ibrutinib, the first BTKi, was approved by the FDA in 2013 for the treatment of chronic lymphocytic leukemia (CLL) and mantle cell lymphoma (MCL) (10). Subsequently, next-generation BTK inhibitors, such as acalabrutinib and pirtobrutinib, have also received FDA approval (10). BTK contains an SH1 kinase domain, with cysteine 481 being the primary target of ibrutinib. Phosphorylation of tyrosine 551 in the kinase domain by SYK or SRC family kinases leads to the autophosphorylation of tyrosine 223 in the SH3 domain, fully activating BTK’s kinase activity (12, 13). While BTK is predominantly located in the cytoplasm, it also shuttles to the nucleus, a process primarily regulated by phosphorylation (14, 15). Under physiological conditions, BTK is essential for cellular proliferation, differentiation, and signaling (16, 17). During periods of extreme cellular stress or invasion of pathogens, BTK can regulate the activation of apoptosis (18). Additionally, BTK can positively regulate its own promoter via the NF-κB pathway, forming an autoregulatory network where BTK not only controls its own transcription but also that of NF-κB, further amplifying its signaling influence (18).

In addition to its established role in tumorigenesis and B-cell proliferation, BTK has been implicated in viral replication processes. *In vivo* studies demonstrate that pharmacological inhibition of BTK exerts protective effects on the lungs during the inflammatory response induced by influenza virus infection, resulting in improved survival rates in mice (19). BTK activity is also heightened in HIV-infected cells, where its suppression enhances viral clearance by promoting the apoptosis of infected cells (20). Moreover, BTK directly phosphorylates Toll-like receptor 3, modulating antiviral responses (21). However, the inhibitory effects of BTK inhibitors (BTKi) on innate and humoral immunity may heighten susceptibility to certain viral infections. For instance, in BTK-deficient macrophages, dengue virus clearance and the secretion of inflammatory cytokines are compromised (21). In severe COVID-19 cases, excessive macrophage activation has been identified as a primary driver of hyperinflammatory responses. Given BTK’s regulatory role in macrophage activity, BTK inhibition has emerged as a therapeutic strategy for COVID-19 infections (22). Clinical observations indicate that patients with CLL and Waldenström macroglobulinemia treated with ibrutinib exhibit only mild symptoms following severe acute respiratory syndrome coronavirus 2 (SARS-CoV-2) infection, suggesting that BTK inhibition may mitigate the inflammatory damage caused by the virus (23). In severe COVID-19 cases, acalabrutinib has been shown to significantly improve oxygenation and reduce inflammatory markers, including C-reactive protein and IL-6, without notable toxicity (22). Additionally, ibrutinib and zanubrutinib may interfere with SARS-CoV-2 viral entry and replication (24). These findings suggest that BTK represents a promising therapeutic target for viral infections, offering both anti-inflammatory and antiviral benefits.

Using high-throughput screening for antivirals against vaccinia virus (VACV), we previously identified ibrutinib as an antiviral candidate, demonstrating significant inhibition of VACV replication in cell culture (25). In this study, we extended our investigation to evaluate the antiviral efficacy of ibrutinib against multiple poxviruses both *in vitro* and *in vivo*. We confirmed that ibrutinib effectively suppressed the replication of MPXV, VACV, and LSDV and provided protective effects against VACV-induced pathogenesis in animal models. Furthermore, genetic knockdown of BTK significantly impaired viral replication, while BTK overexpression enhanced it. During poxvirus infection, BTK phosphorylation was markedly increased, and the kinase translocated to the nucleus. Mutations in key phosphorylation sites of BTK attenuated its pro-viral effects on VACV’s replication. Collectively, our findings demonstrate that ibrutinib is a promising antiviral agent against multiple poxviruses, exerting its effects by targeting BTK, a newly identified factor that can promote poxvirus replication.

## RESULTS

### Ibrutinib effectively inhibits the replication of VACV in cell culture

To validate the findings from the high-throughput screening (25), we first assessed the antiviral activity of ibrutinib across several human cell lines, including HeLa, A549, and THP-1 cells. These cells were infected with the VACV-WR (Western Reserve) strain at either 3 or 0.01 pfu/cell. At the same time, cells were treated with ibrutinib at varying concentrations, and dimethyl sulfoxide (DMSO) served as a negative control. Additionally, uninfected cells treated solely with ibrutinib were included to evaluate the drug’s impact on cell viability.

**Fig 1:**
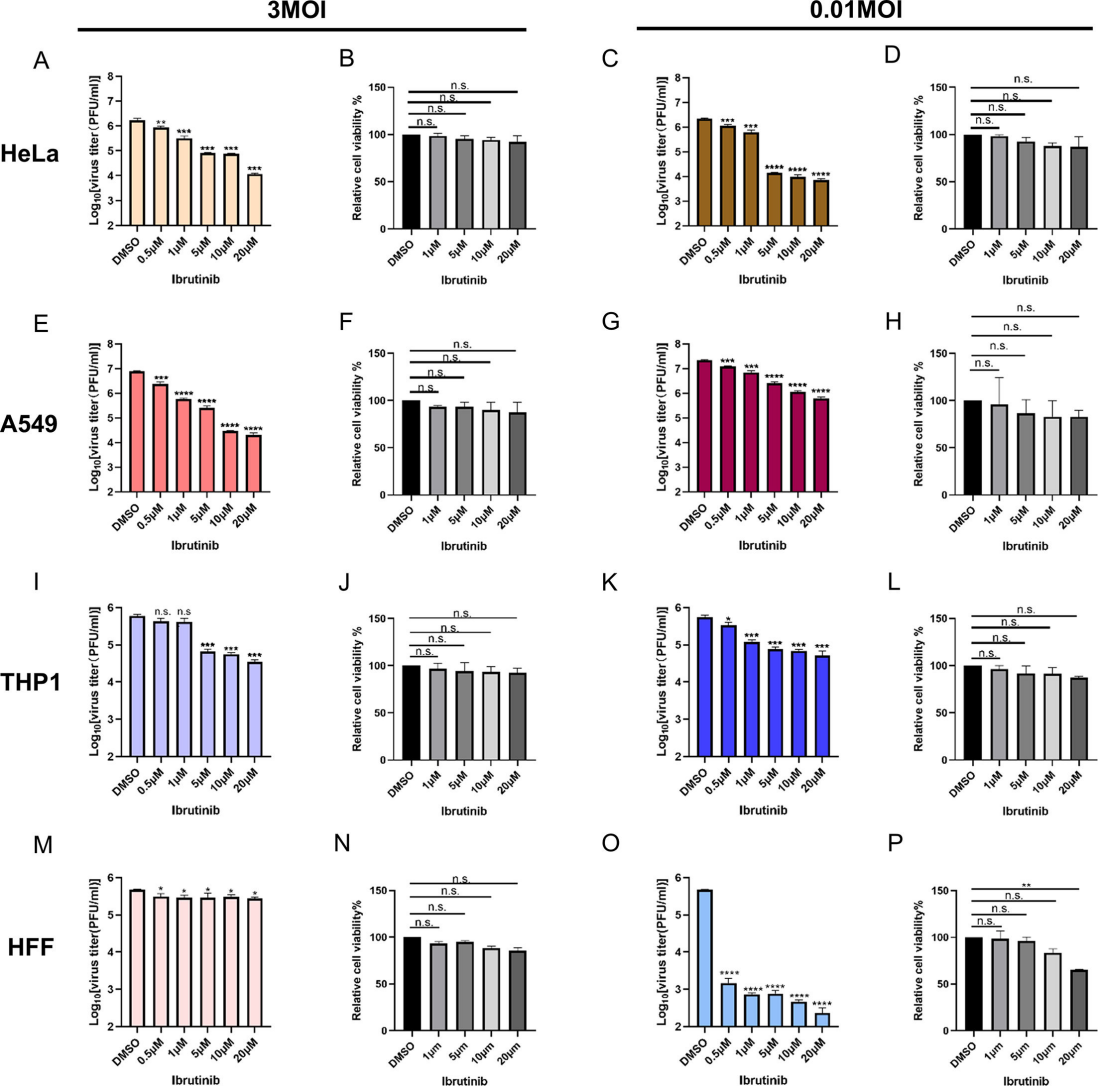
Ibrutinib inhibits VACV replication in different human cells. (**A**) HeLa, (**E**) A549, (**I**) THP-1, and HFF (**M**) cells were treated with ibrutinib at the concentrations mentioned above while cells were infected with VACV-WR at 3 pfu/cell. Viruses were collected at 24 hours post-infection (hpi) and titered in BS-C-1 cells. (**C**) HeLa, (**G**) A549, (**K**) THP1, and HFF (**O**) cells were treated with ibrutinib at the concentrations mentioned above while cells were infected with VACV-WR at 0.01 pfu/cell. Viruses were collected at 48 hpi and titered in BS-C-1 cells. (**B**) HeLa, (**F**) A549, (**J**) THP1, and HFF (**N**) cells were treated with ibrutinib at the concentrations mentioned above for 24 h, and cell viability was measured by an MTT assay. (**D**) HeLa, (**H**) A549, (**L**) THP1, and HFF (**P**) cells were treated with ibrutinib at the concentrations mentioned above for 48 h, and cell viability was measured by an MTT assay. Data (**A–P**) were collected from three independent experiments, and they are presented as mean ± standard deviation. One-way analysis of variance (ANOVA) was used to analyze the data. *P < 0.05, **P < 0.01, ***P < 0.001, and ****P < 0.0001; n.s., non-significant.

In HeLa and A549 cells, a notable antiviral effect was observed at a concentration of 0.5 µM, with a dose-dependent inhibition of viral replication detected at both high and low multiplicities of infection (MOIs) (Fig. 1A, C, E, and G). At 20 µM, ibrutinib significantly reduced viral titers by approximately 100–1,000× fold. However, in THP1 cells infected at a high MOI, the antiviral effect of ibrutinib was not observed until the concentration reached 5 µM. In contrast, in a low MOI infection, ibrutinib at 0.5 µM exhibited a moderate antiviral effect against the VACV infection (Fig. 1I and K). In human foreskin fibroblasts (HFF) cells, ibrutinib exhibited a stronger antiviral effect during low MOI infection compared to high MOI infection (Fig. 1M and O). In addition, cell viability was monitored upon ibrutinib treatment, and no significant reduction in cell viability was observed in HeLa, A549, THP-1, and HFF cells (Fig. 1B, D, F, H, J, L, and N). In contrast, ibrutinib treatment at 20 µM showed significant cytotoxicity in HFF cells (Fig. 1P). However, since antiviral effects were observed at lower concentrations that did not affect cell viability, these data support that the antiviral activity of ibrutinib is not attributable to drug-induced cytotoxicity.

### Ibrutinib exhibits broad-spectrum inhibition on virus replication

**Fig 2:**
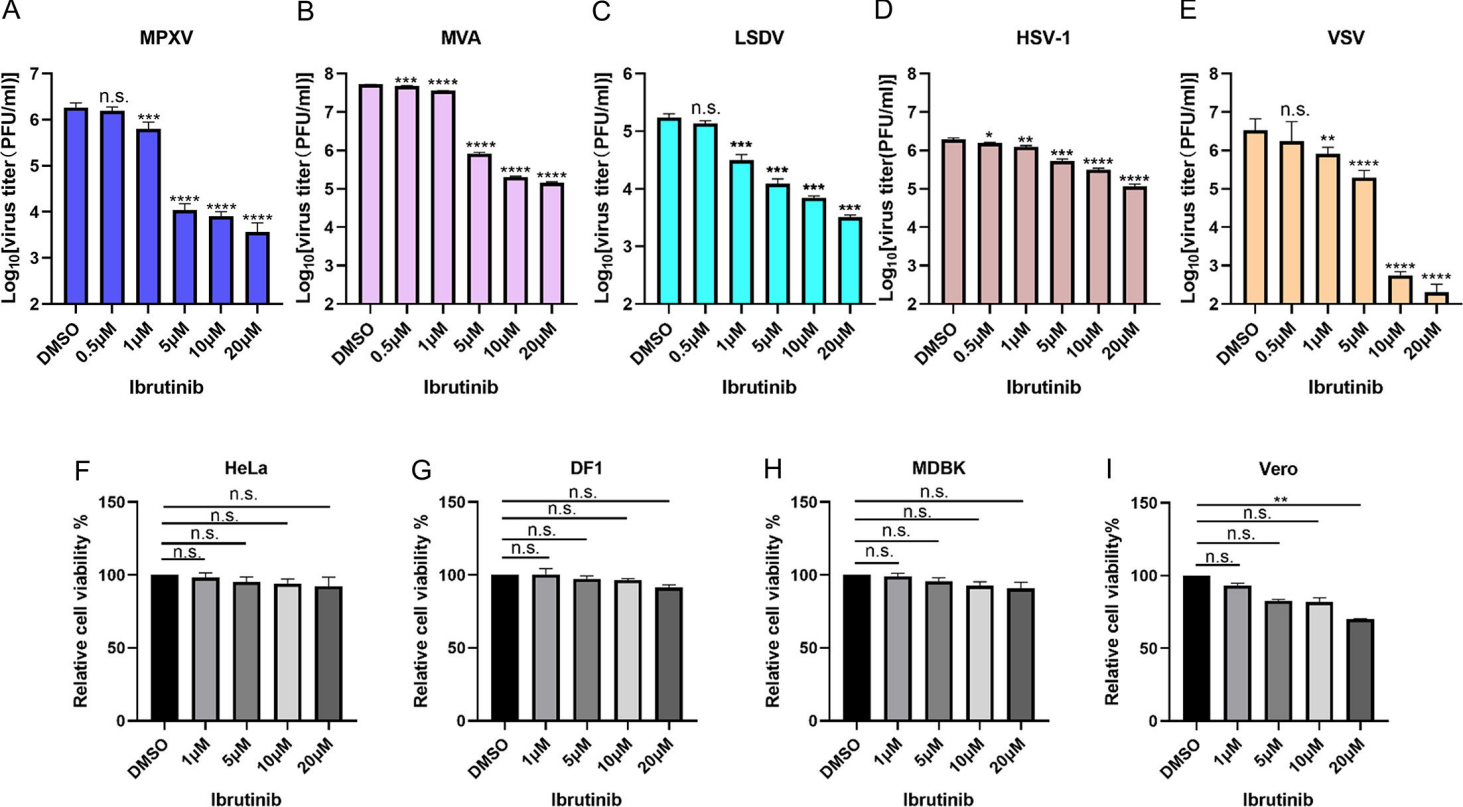
Ibrutinib inhibits replication of mpox, MVA, LSDV, HSV-1, and VSV. (**A**) HeLa cells were treated with ibrutinib at the concentrations mentioned above while cells were infected with mpox at 3 pfu/cell. Viruses were collected at 24 hpi and titered in BS-C-1 cells. (**B**) DF-1 cells were treated with ibrutinib at the concentrations mentioned above while cells were infected with MVA at 3 pfu/cell. Viruses were collected at 24 hpi and titered in DF-1 cells. (**C**) MDBK cells were treated with ibrutinib at the concentrations mentioned above while cells were infected with LSDV at 3 pfu/cell. Viruses were collected at 48 hpi and titered in MDBK cells. (**D**) Vero cells were treated with ibrutinib at the concentrations mentioned above while cells were infected with HSV-1 at 3 pfu/cell. Viruses were collected at 24 hpi and titered in Vero cells. (**E**) HeLa cells were treated with ibrutinib at the concentrations mentioned above, while cells were infected with VSV at 3 pfu/cell, the titer of VSV was determined by the TCID50 method. (**F**) HeLa cells were treated with ibrutinib at the concentrations mentioned above for 24 h, and cell viability was measured by an MTT assay. (**G**) DF-1 cells were treated with ibrutinib at the concentrations mentioned above for 24 h, and cell viability was measured by an MTT assay. (**H**) MDBK cells were treated with ibrutinib at the concentrations mentioned above for 48 h, and cell viability was measured by an MTT assay. (**I**) Vero cells were treated with ibrutinib at the concentrations mentioned above for 24 h, and cell viability was measured by an MTT assay. Data (**A–I**) were collected from three independent experiments, and they are presented as mean ± standard deviation. One-way ANOVA was used to analyze the data. **P < 0.01, ***P < 0.001, and ****P < 0.0001; n.s., non-significant.

To examine if ibrutinib displays any broad-spectrum inhibitory effect on virus replication, four more DNA viruses, including MPXV, modified vaccinia virus Ankara (MVA), LSDV, and herpes simplex 1 virus (HSV-1), as well as one RNA virus, vesicular stomatitis virus (VSV), were tested. HeLa, DF-1, and MDBK cells were infected with MPXV, MVA, and LSDV, respectively, at 3 pfu/cell, while increasing dosages of ibrutinib were added to the cells at the time of infection. Viruses were harvested at 24 h and subjected to quantitation using the plaque assay. Results showed that ibrutinib exhibited a potent inhibitory effect on the replication of MPXV, a member of the orthopoxviruses, and MVA, an approved smallpox and mpox vaccine (Fig. 2A and B). In addition, the replication of LSDV in MDBK cells was inhibited by ibrutinib in a dose-dependent manner (Fig. 2C). These data indicated that ibrutinib exhibited a broad-spectrum inhibition on multiple poxviruses.

Next, we aimed to investigate whether ibrutinib was able to inhibit viruses other than poxviruses. Vero and HeLa cells were infected with HSV-1 and VSV, respectively, at 3 pfu/cell, while increasing dosages of ibrutinib were added to the cells. Viruses were harvested at 24 h, and virus titers were detected by plaque assay. The results demonstrated that while ibrutinib inhibited the replication of HSV-1, its effect was less potent compared to its impact on poxviruses (Fig. 2D). Nevertheless, the replication of VSV was inhibited by ibrutinib in a dose-dependent manner (Fig. 2E). In addition, in most of the cell lines tested, ibrutinib did not demonstrate cell toxicity with the exception of Vero cells, in which ibrutinib at 20 µM significantly reduced cell viability (Fig. 2F through I).

### Ibrutinib targets viral DNA replication and post-replicative gene expression

**Fig 3:**
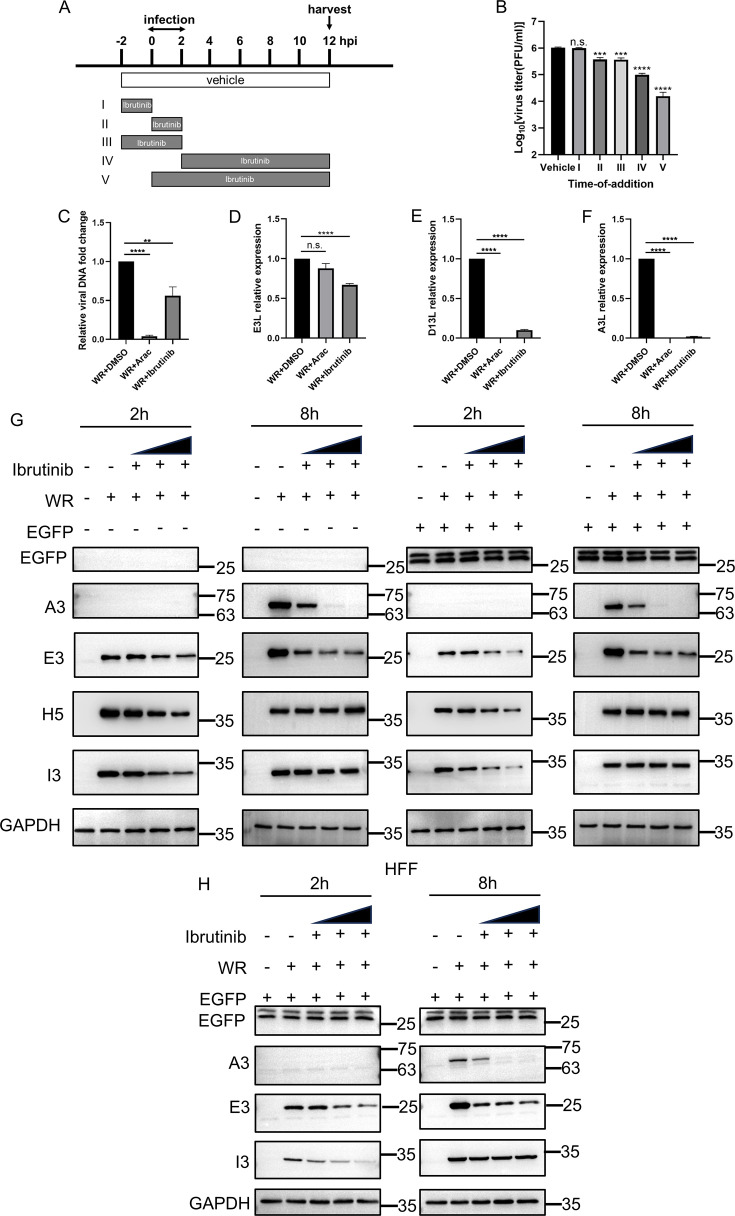
Ibrutinib acted in the VACV-WR infection cycle. (**A**) Strategy of the time-of-drug-addition assay. All samples were infected with the VACV-WR between 0 and 2 h, and the course of ibrutinib addition was set into five intervals (I–V). (**B**) Viral titers of VACV-WR were quantified via plaque assay. (**C**) HeLa cells were treated with DMSO, Arac (40 µg/mL), or indicated concentrations of ibrutinib (10 µM). Viral DNA was harvested at 8 hpi, and relative viral DNA copy numbers in cell lysates were tested for each sample using quantitative PCR (qPCR). (**D**) Total RNA was harvested at 2 hpi, and the relative expression of E3L was detected by qPCR. (**E**) Total RNA was harvested at 8 hpi, and the relative expression of D13L was detected by qPCR. (**F**) Total RNA was harvested at 8 hpi, and the relative expression of A3L was detected by qPCR. (**G**) HeLa cells were transfected with pMLS-SV40-EGFP plasmids at 1.5 µg for 24 h or left untransfected, and the cells were then infected with 3 pfu/cell WR for 2 or 8 h, and ibrutinib was added at the same time of infection (1, 5, and 10 µM), and the expressions of EGFP, E3, H5, I3, and A3 proteins were detected by western blot. (**H**) HFF cells were transfected with pMLS-SV40-EGFP plasmids at 1.5 µg for 24 h, and the cells were infected with 3 pfu/cell WR for 2 or 8 h, and ibrutinib was added at the same time of infection (1, 5, and 10 µM), and the expressions of EGFP, E3, I3, and A3 proteins were detected by western blot.

Poxviruses replicate in a highly regulated temporal sequence, with gene expression following a precise and ordered progression. Early gene expression is initiated immediately upon viral entry into the host cell, followed by the replication of the viral DNA. Importantly, intermediate and late gene expression are contingent upon successful DNA replication, ensuring that the replication process is tightly controlled and coordinated (26). To assess the specific stage of the viral replication cycle targeted by ibrutinib, we performed a time-of-addition assay in HeLa cells infected with VACV. Cells were infected with VACV-WR at 3 pfu/cell, and ibrutinib was added at 10 µM at different time points during the experiment (illustrated in Fig. 3A). Although no antiviral activity was observed for the pre-treatment-only condition (I), we observed robust inhibition of virus titers when ibrutinib was present throughout the experiment or when it was added after the inoculation period (Fig. 3B). These results suggested that ibrutinib most likely affected a post-entry stage of the viral replication cycle.

To further dissect the steps affected by ibrutinib, viral mRNA transcription, protein synthesis, as well as DNA levels were monitored upon ibrutinib treatment. Cytosine arabinoside (AraC) was included as a positive control, which inhibits viral DNA replication and post-replicative gene expression without affecting viral early protein synthesis. HeLa cells were infected by VACV-WR at 3 pfu/cell in the presence of DMSO, AraC (40 µg/mL), or ibrutinib (10 µM). Viral DNA was harvested at 8 hours post-infection (hpi), and total RNA was harvested at 2 and 8 hpi. Early gene (E3L) was determined by quantitative PCR (qPCR) at 2 hpi, and relative viral DNA levels and viral intermediate (D13L) and late gene (A3L) levels in infected cells were determined by qPCR at 8 hpi. Results from the qPCR demonstrated that viral DNA levels were reduced to 50% upon ibrutinib treatment (Fig. 3C). In addition, ibrutinib exhibited a moderate effect on viral early mRNA abundance (E3L) but almost completely abolished viral intermediate (D13L) and late gene (A3L) transcription (Fig. 3D through F). The synthesis of selected viral proteins was detected by western blotting analysis following viral infection in HeLa cells. VACV E3, H5, and I3, three early proteins, were inhibited by ibrutinib treatment, and the synthesis of A3, a viral late protein, was strongly inhibited (Fig. 3G). Notably, the inhibitory effect of ibrutinib on H5 and I3 was no longer observed at 8 h (Fig. 3G). A similar result was observed in HFF cells (Fig. 3H). To verify if the effect on protein synthesis was specific to viral proteins, enhanced GFP (eGFP) directed by the SV40 promoter was transfected, and the synthesis of eGFP was not affected by the presence of ibrutinib, suggesting the inhibitory effect was specific to viral proteins (Fig. 3G and H). Overall, these data demonstrated that ibrutinib reduced viral DNA levels and post-replicative protein synthesis.

### Ibrutinib mitigates the pathogenic effects of VACV *in vivo*

**Fig 4:**
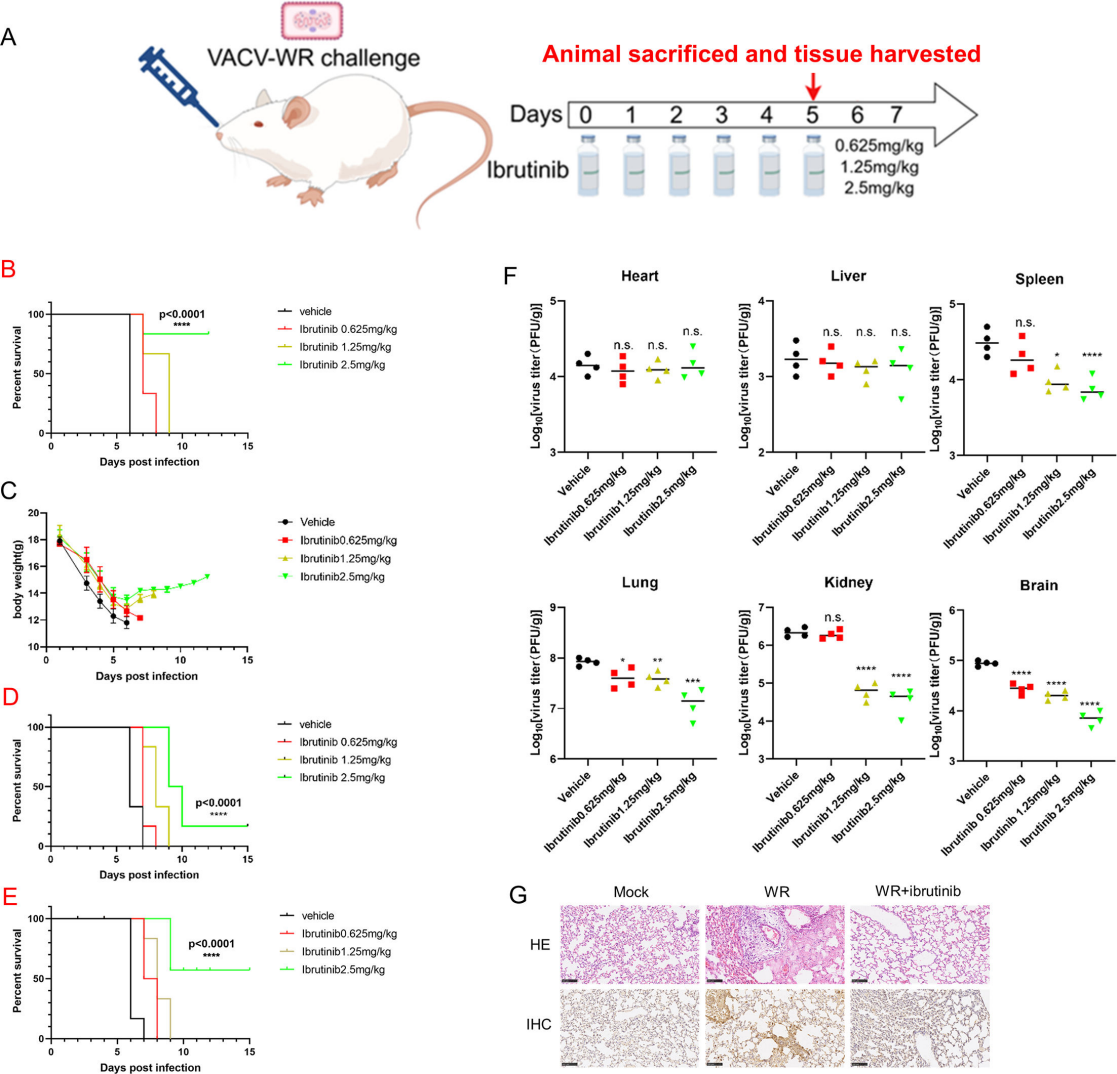
Ibrutinib prolonged the survival of VACV-infected mice and decreased viral abundance in various organs. (**A**) Schematic diagram of VACV-WR challenge and sample collection. (**B**) Survival curves. Seven- to eight-week-old female BALB/c mice were divided into four groups, and six female BALB/c mice were included in each group. BALB/c mice were infected with VACV-WR at 2 × 10^4^ pfu in the presence of ibrutinib at the above concentrations or phosphate-buffered saline. Statistical comparisons in the survival curves were performed using the log-rank test. (**C**) Weight curves. Throughout the experimental period, mouse weight was recorded daily for a maximum of 14 days. (D–E) Survival curves. The same experimental procedures as described in Fig. 4B were followed. Statistical comparisons of survival curves were performed using the log-rank test. (**F**) Viral loads. At 5 days post-infection, the viral yield in mouse hearts, livers, spleens, lungs, kidneys, and brain was quantified via plaque assay. (**G**) H&E staining and immunohistochemistry assay of sectioned lungs. At 2 days post-infection, pathological changes were evaluated based on neutrophil infiltration and necrosis using H&E staining. The viral antigens were visualized using VACV-antibodies via an immunohistochemistry assay. Yellow staining is considered a viral antigen. Statistical comparisons were performed using one-way ANOVA (*P < 0.05, **P < 0.01, ***P < 0.001, and ****P < 0.0001; n.s., non-significant).

Next, we aimed to determine the antiviral effect of ibrutinib *in vivo*. To do this, we employed a mouse model in which intranasal challenge with VACV leads to significant weight loss and eventual death in BALB/c mice (27), accompanied by pronounced pathogenic lesions in the lungs (Fig. 4A). A prospective study of ibrutinib in 63 symptomatic patients with Waldenström’s macroglobulinemia who had received at least one previous treatment was performed, and ibrutinib at a daily dose of 420 mg was administered orally until disease progression or the development of unacceptable toxic effects (28). In addition, relevant literature previously reported that the dosage of ibrutinib for C57BL/6J mice (aged 7–8 weeks and weighing 16.8–19.9 g) was 2.6 mg/kg (19). In our study, six female BALB/c mice in each group were infected intranasally with the VACV-WR strain at 2 × 10^4^ pfu, followed by daily ibrutinib treatment at three different dosages (0.625, 1.25, and 2.5 mg/kg), with phosphate-buffered saline (PBS) serving as the negative control. Animals were monitored until either death or a weight loss exceeding 30%, at which point they were euthanized and recorded as deceased. All PBS-treated mice succumbed within 6 days of inoculation, showing substantial weight loss from the first day of infection (Fig. 4B and C). In contrast, ibrutinib treatment at a dose of 2.5 mg/kg significantly prolonged survival (*P* < 0.0001) and delayed virus-induced weight loss (Fig. 4B and C). In order to further solidify our conclusion and demonstrate the effectiveness of ibrutinib, we conducted two additional mouse survival experiments. Ibrutinib treatment at a dose of 2.5 mg/kg significantly prolonged the survival of infected mice, although the number of mice that ultimately survived was different (Fig. 4D and E). Viral loads in the heart, liver, spleen, lungs, kidneys, and brain were quantified 5 days post-infection via plaque assay. As illustrated in Fig. 4F, while viral loads in the heart and liver of ibrutinib-treated animals were comparable to those of the PBS-treated group, the viral loads in the spleen, lungs, kidneys, and brain were significantly reduced in a dose-dependent manner. Histological examination of lung tissues on day 2 post-infection revealed neutrophil infiltration and necrosis with marked inflammation. In comparison, ibrutinib treatment attenuated infection-induced pathological lesions in the lungs. Immunohistochemical analysis using anti-VACV antibodies further confirmed reduced viral antigen presence in the ibrutinib-treated group compared to controls (Fig. 4G). In summary, ibrutinib demonstrated a clear inhibitory effect on VACV-WR-induced pathogenesis, prolonged survival, and alleviated virus-induced pathogenic conditions in infected mice.

### Inhibition of BTK functionality suppresses VACV replication

**Fig 5:**
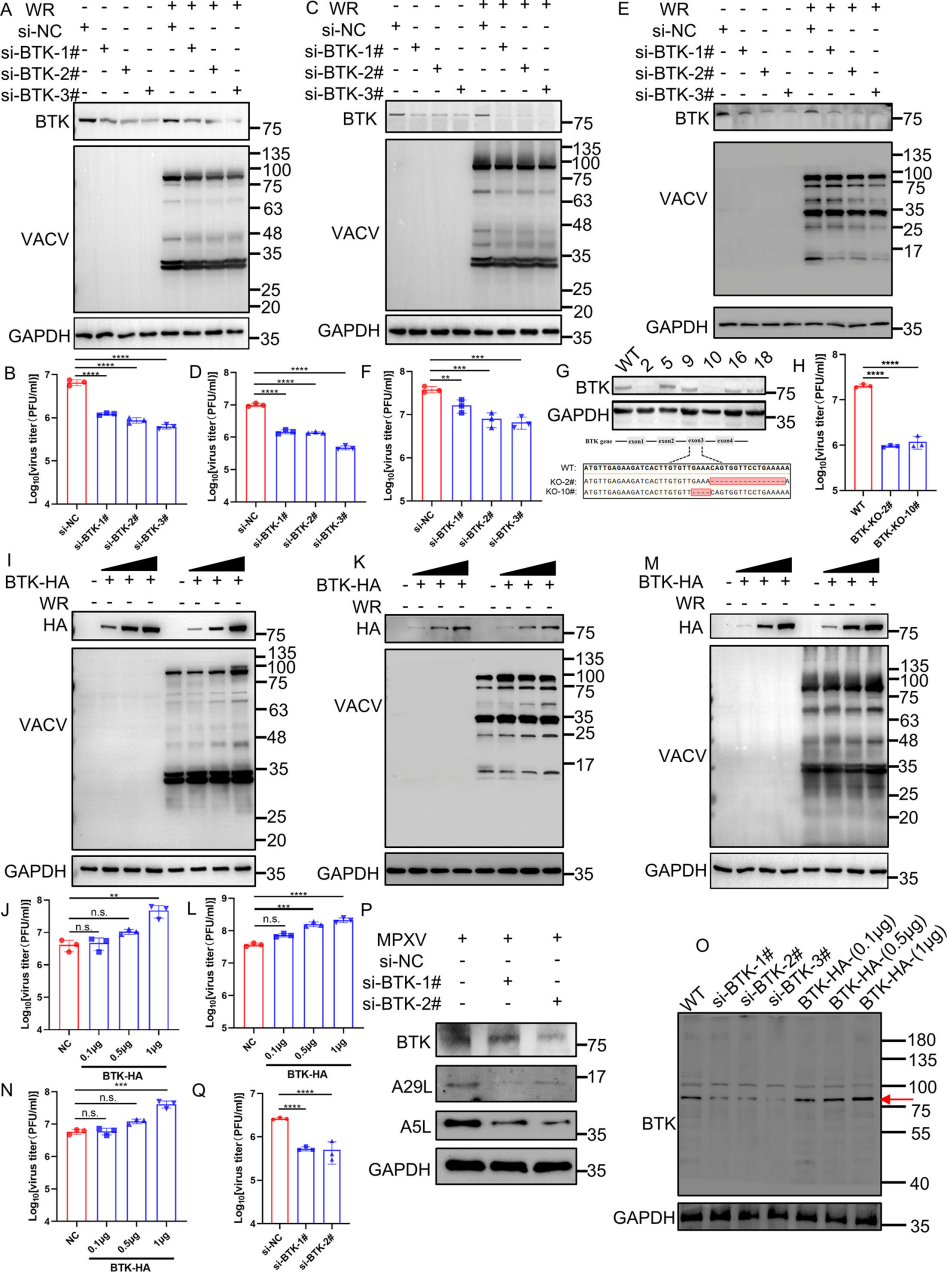
Impact of BTK knockdown and overexpression on VACV-WR and MPXV infection. (**A**) HeLa cells were transfected with BTK siRNA (80 nM) and negative control siRNA for 48 h, then were infected with VACV-WR at 0.01 pfu/cell for 48 h. Samples were lysed and proteins resolved by SDS-PAGE and western blotting with anti-BTK, anti-VACV antiserum, and anti-GAPDH antibodies. VACV: anti-VACV antiserum. (**B**) HeLa cells were transfected with BTK siRNA (80 nM) and negative control siRNA for 48 h, then were infected with VACV-WR at 0.01 pfu/cell for 48 h. The whole cell lysates were collected and titered in BS-C-1 cells. (**C**) A549 cells were transfected with BTK siRNA (80 nM) and negative control siRNA for 48 h, then were infected with VACV-WR at 0.01 pfu/cell for 48 h. The whole cell lysates were collected and proteins were resolved by SDS-PAGE and western blotting with anti-BTK, anti-VACV antiserum, and anti-GAPDH antibodies. VACV: anti-VACV antiserum. (**D**) A549 cells were transfected with siBTK (80 nM) and negative control siRNA for 48 h, then were infected with VACV-WR at 0.01 pfu/cell. The whole cell lysate was collected at 48 hpi, and viruses within were titered in BS-C-1 cells. (**E**) HFF cells were transfected with BTK siRNA (80 nM) and negative control siRNA for 48 h, then were infected with VACV-WR at 0.01 pfu/cell for 48 h. Samples were lysed and proteins resolved by SDS-PAGE and western blotting with anti-BTK, anti-VACV antiserum, and anti-GAPDH antibodies. VACV: anti-VACV antiserum. (**F**) HFF cells were transfected with BTK siRNA (80 nM) and negative control siRNA for 48 h, then were infected with VACV-WR at 0.01 pfu/cell for 48 h. Viruses in whole cell lysate were collected and titered in BS-C-1 cells. (**G**) BTK-KO cells (2#,10#) were generated by CRISPR-Cas9 technology. (**H**) BTK-KO cells were infected with 3 pfu/cell WR for 24 h, and then the whole cell lysates were collected and viruses within were titered in BS-C-1 cells. (**I**) HeLa cells were transfected with BTK plasmids at 0.1, 0.5, and 1 µg for 24 h, then were infected with VACV-WR at 0.01 pfu/cell for 48 h. The whole cell lysates were collected, and proteins were lysed and resolved by SDS-PAGE and western blotting with anti-BTK, anti-VACV antiserum, and anti-GAPDH antibodies. VACV: anti-VACV antiserum. (**J**) HeLa cells were transfected with BTK plasmids at 0.1, 0.5, and 1 µg for 24 h, then were infected with VACV-WR at 0.01 pfu/cell. The whole cell lysates were collected at 48 hpi and viruses within were titered in BS-C-1 cells. NC: negative control. (**K**) HFF cells were transfected with BTK plasmids at 0.1, 0.5, and 1 µg for 24 h, then were infected with VACV-WR at 0.01 pfu/cell for 48 h. Samples were lysed and proteins resolved by SDS-PAGE and western blotting with anti-BTK, anti-VACV antiserum, and anti-GAPDH antibodies. VACV: anti-VACV antiserum. (**L**) HFF cells were transfected with BTK plasmids at 0.1, 0.5, and 1 µg for 24 h, then were infected with VACV-WR at 0.01 pfu/cell. Viruses in whole cell lysate were collected at 48 hpi and titered in BS-C-1 cells. NC: negative control. (**M**) A549 cells were transfected with BTK plasmid at 0.1, 0.5, and 1 µg for 24 h, then were infected with VACV-WR at 0.01 pfu/cell for 48 h. The whole cell lysates were collected, and proteins were lysed and resolved by SDS-PAGE and western blotting with anti-BTK, anti-VACV antiserum, and anti-GAPDH antibodies. VACV: anti-VACV antiserum. (**N**) A549 cells were transfected with BTK plasmids at 0.1, 0.5, and 1 µg for 24 h, then were infected with VACV-WR at 0.01 pfu/cell. The whole cell lysates were collected at 48 hpi and viruses within were titered in BS-C-1 cells. NC: negative control. (**O**) HFF cells were transfected with BTK siRNA or BTK plasmids at 0.1, 0.5, and 1 µg for 48 h. Samples were lysed and proteins resolved by SDS-PAGE and western blotting with anti-BTK antibody. (**P and Q**) HeLa cells were transfected with BTK siRNA (80 nM) and negative control siRNA, and then the cells were infected with MPXV at 3 pfu/cell. At 24 hpi, the expression of viral proteins in infected cells was detected by western blotting using the indicated antibodies (Fig. 5P), and virus titers were determined by a plaque assay (Fig. 5Q). Data (**A–F, H–N, P and Q**) were collected from three independent experiments, and they are presented as mean ± standard deviation. One-way ANOVA followed by Tukey’s multiple comparisons test was used to analyze the data. Statistics: n.s., not significant; *P* > 0.05, **P < 0.01, ***P < 0.001, and ****P < 0.0001.

Ibrutinib is known to effectively block the phosphorylation and activation of BTK in mammalian cells (29). To assess whether ibrutinib inhibits poxvirus replication by targeting BTK, we first manipulated BTK levels in HeLa cells and examined its impact on viral replication. Cells were transfected with BTK-targeting small interfering RNA (siRNA) (siBTK) or a negative control (siNC) for 48 h, followed by infection with VACV-WR at 0.01 pfu/cell for an additional 48 h. Viral titers were quantified via plaque assay in BS-C-1 cells. Notably, siBTK transfection dramatically reduced BTK levels, resulting in a moderate reduction in viral protein synthesis (Fig. 5A) and up to a 10-fold reduction in virus yield (Fig. 5B). Similar findings were observed in A549 cells (Fig. 5C and D) and HFF cells (Fig. 5E and F). Next, BTK-KO HeLa cells were generated by CRISPR-Cas9 technology, and viral replication was examined in the clones with successful BTK depletion (Fig. 5G). Deletion of BTK exhibited reduced virus replication, confirming that BTK facilitates the replication of VACV (Fig. 5H). To further confirm the role of BTK in virus replication, HeLa cells were transfected with BTK, followed by viral infection, and ectopic expression of human BTK promoted both viral protein synthesis and viral titers in a dose-dependent manner (Fig. 5I and J). Similar findings were observed in HFF cells (Fig. 5K and L). In addition, ectopic expression of human BTK in A549 cells through transient transfection led to a dose-dependent increase in both viral protein synthesis and viral titers, further supporting BTK’s role in promoting viral replication (Fig. 5M and N). The specificity of the antibody used for BTK was examined (Fig. 5O).

We also explored BTK’s involvement in MPXV infection using a similar protocol. While siBTK reduced viral protein synthesis and titers in HeLa cells, the reduction in MPXV viral load was significantly less in cells with BTK knockdown than that in control cells, although less pronounced than in VACV-infected cells (Fig. 5P and Q). Collectively, these findings indicate that BTK contributes to the replication of both VACV and MPXV in mammalian cells.

### Phosphorylation of BTK is essential for the promotion of viral replication

**Fig 6:**
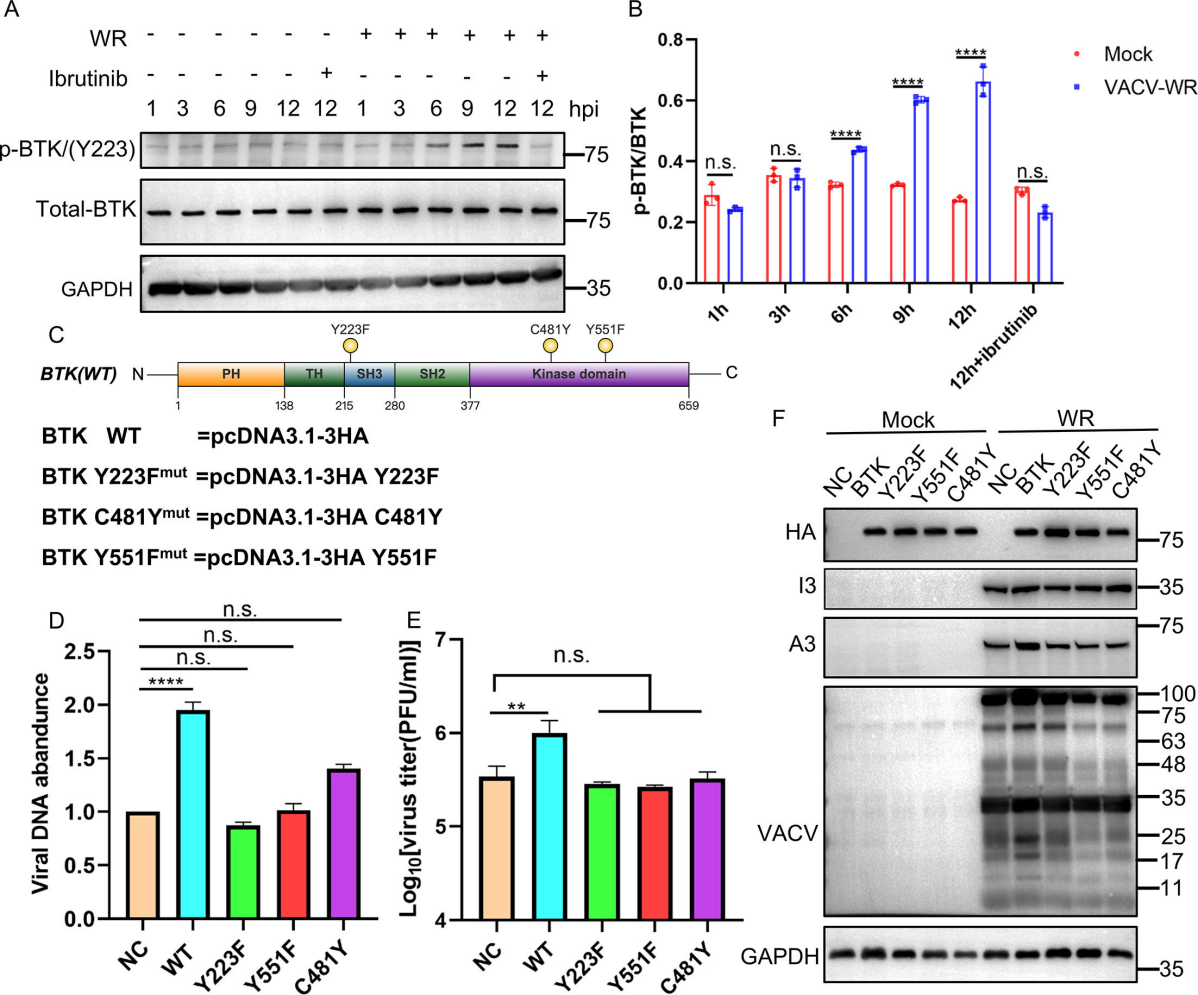
BTK was phosphorylated upon VACV-WR infection, and point mutations of BTK exhibited decreased ability to promote viral replication. (**A**) HeLa cell lines were either uninfected or infected with VACV-WR at 3 pfu/cell in the presence or absence of ibrutinib, and samples were harvested at the specified time. Phosphorylation levels of BTK protein and total BTK protein levels were determined via western blotting. (**B**) The ratio of phosphorylated BTK to total BTK protein level was measured and calculated by ImageJ. (**C**) Schematic diagram of BTK and its mutants. (**D**) HeLa-BTK-KO cells in triplicate were transfected with BTK and BTK mutants for 24 h, and then were infected with 3 pfu/cell WR for 6 h, and samples were collected. E11L was detected by qPCR to detect the effect of mutants on DNA levels. NC: negative control (**E**) BTK and BTK mutants were transfected into HeLa-BTK-KO cells in triplicate for 24 h, and the cells were infected with 0.01 pfu/cell WR, and viruses were collected at 48 hpi and titered in BS-C-1 cells. NC: negative control (**F**) BTK and BTK mutants were transfected into HeLa-BTK-KO cells for 24 h, and then the cells were infected with 0.01 pfu/cell WR for 24 h. The expression of viral proteins was detected by western blotting analysis after the samples were collected. NC, negative control; VACV, anti-VACV antiserum.

Since BTK activation requires phosphorylation at specific tyrosine residues, such as Y223, we investigated whether VACV infection triggers BTK phosphorylation. HeLa cells were infected with VACV-WR at 3 pfu/cell for 12 h, and proteins were harvested to assess BTK phosphorylation using a phospho-specific antibody targeting Y223. While total p-BTK levels remained unchanged in uninfected cells, VACV infection led to an evident increase in Y223 phosphorylation starting at 3 hpi, peaking by 12 hpi, and the addition of ibrutinib inhibited BTK phosphorylation at 12 hpi. The ratios between phosphorylated BTK and total BTK were measured and calculated by ImageJ (Fig. 6A and B). Phosphorylation at Y551 and Y223 is known to be critical for BTK activation, and ibrutinib inhibits this process by covalently binding to C481, thereby blocking its kinase activity (30). To further examine the role of these sites in viral replication, we generated three BTK mutants by substituting Y223, C481, and Y551 with phenylalanine or tyrosine (Fig. 6C). HeLa cells were transfected with either wild-type (WT)-BTK or the mutants for 24 h, followed by infection with VACV-WR at 3 pfu/cell. Quantification of viral DNA and total virus yield revealed that while WT-BTK enhanced viral DNA levels and overall viral replication, the BTK mutants failed to do so (Fig. 6D and E). Additionally, viral protein synthesis, assessed by anti-VACV antiserum, exhibited a similar pattern (Fig. 6F). These findings underscore the importance of BTK phosphorylation and ibrutinib’s target sites for BTK’s pro-viral function.

### VACV induces the nuclear translocation of BTK

**Fig 7:**
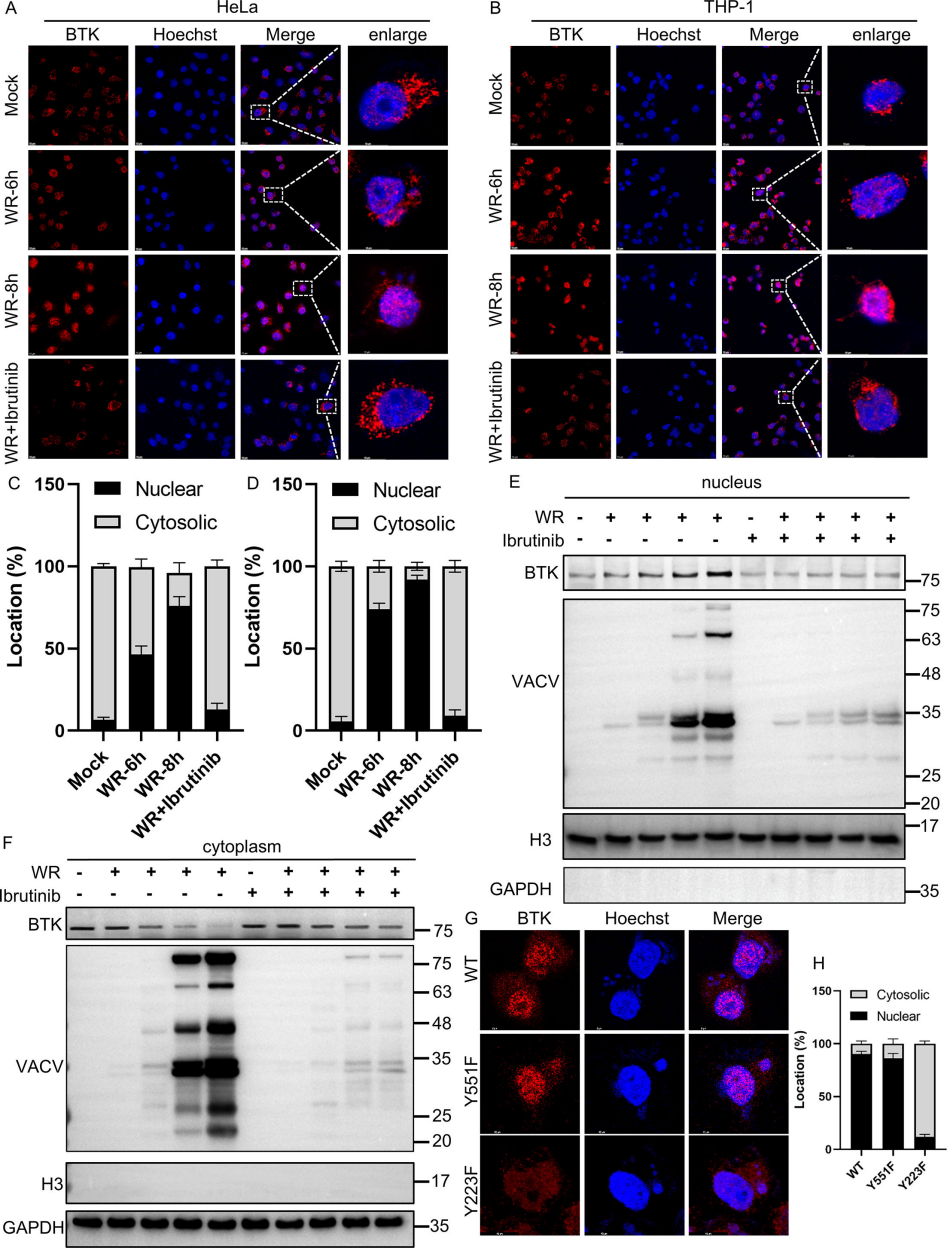
During virus infection, BTK can enter the nucleus. (**A**) HeLa and (**B**) THP-1 cells were either untreated or infected with VACV at 3 pfu/cell for the indicated time in the presence or absence of ibrutinib (10 µM). A confocal experiment was performed to detect the subcellular localization of BTK. (**C**) The confocal analyses were performed in triplicate, and the localization of BTK was quantified in 50 randomly selected HeLa cells, and bars represent mean values ± SD. (**D**) The confocal analyses were performed in triplicate, and the localization of BTK was quantified in 50 randomly selected THP-1 cells, and bars represent mean values ± SD. (**E and F**) HeLa cells were infected with VACV-WR at 3 pfu/cell for the indicated time in the presence or absence of ibrutinib (10 µM), and then nuclear and cytoplasmic proteins were extracted using a nucleoplasmic separation kit and subjected to western blotting analysis with anti-BTK, anti-VACV antiserum, anti-histone H3 (H3 stands for Histone H3, a housekeeping gene of the nucleus), and anti-GAPDH antibodies. VACV: anti-VACV antiserum. (**G**) HeLa cells were infected with VACV-WR for 2 h and then were transfected with BTK and its mutants (WT, Y551F, and Y223F) for 24 h. After that, a confocal experiment was performed to detect the subcellular localization of BTK and its mutants. (**H**) The confocal microscopic analysis was performed in triplicate, and the localization of BTK was quantified in 50 randomly selected HeLa cells, and bars represent mean values ± SD.

Although the nuclear translocation of BTK is not required for its activation as BTK primarily propagates downstream signaling pathway within the cytoplasm, recent reports suggested that BTK may also shuttle to the nucleus under certain conditions, such as DNA damage response (31). To assess if VACV infection alters the subcellular localization of BTK, we monitored BTK by confocal microscopic analysis upon VACV infection at 6 or 8 hpi in HeLa and THP-1 cells. In mock-infected cells, BTK was primarily observed in the cytoplasm, while VACV infection led to an evident translocation of the protein to the nucleus. However, the treatment of ibrutinib inhibited the nucleus shuttling (Fig. 7A). Similar results were also observed in THP-1 cells (Fig. 7B). The subcellular localization of BTK in HeLa (Fig. 7C) and THP-1 (Fig. 7D) cells upon viral infection was also quantified. Additionally, the nuclear translocation of BTK was confirmed by the separation of cytoplasmic and nuclear parts, followed by western blotting analysis (Fig. 7E and F). Previous studies reported that phosphorylation sites of BTK contributed to its nuclear translocation (18). Given that increased BTK-Y223 phosphorylation and nucleus translocation of BTK were observed during VACV-WR infection, we hypothesized that Y223 and Y551 of BTK may affect its nucleus translocation. To test this, HeLa cells were transfected with WT-BTK or its mutants (Y551F and Y223F) for 24 h, and then infected with VACV-WR at 3 pfu/cell for 8 h. Subcellular localization of BTK was observed by confocal microscopy analysis. Although Y551F did not alter the localization of BTK in comparison to WT-BTK, Y223F led to a reduction of nuclear accumulation of the protein (Fig. 7G). The subcellular localization of BTK and its mutants upon viral infection was also quantified, and the results are shown in Fig. 3H. Overall, these data demonstrated that VACV infection led to the nuclear translocation of BTK, and the Y223 residue was essential for this process.

### Ibrutinib treatment primes the type I interferon response

**Fig 8:**
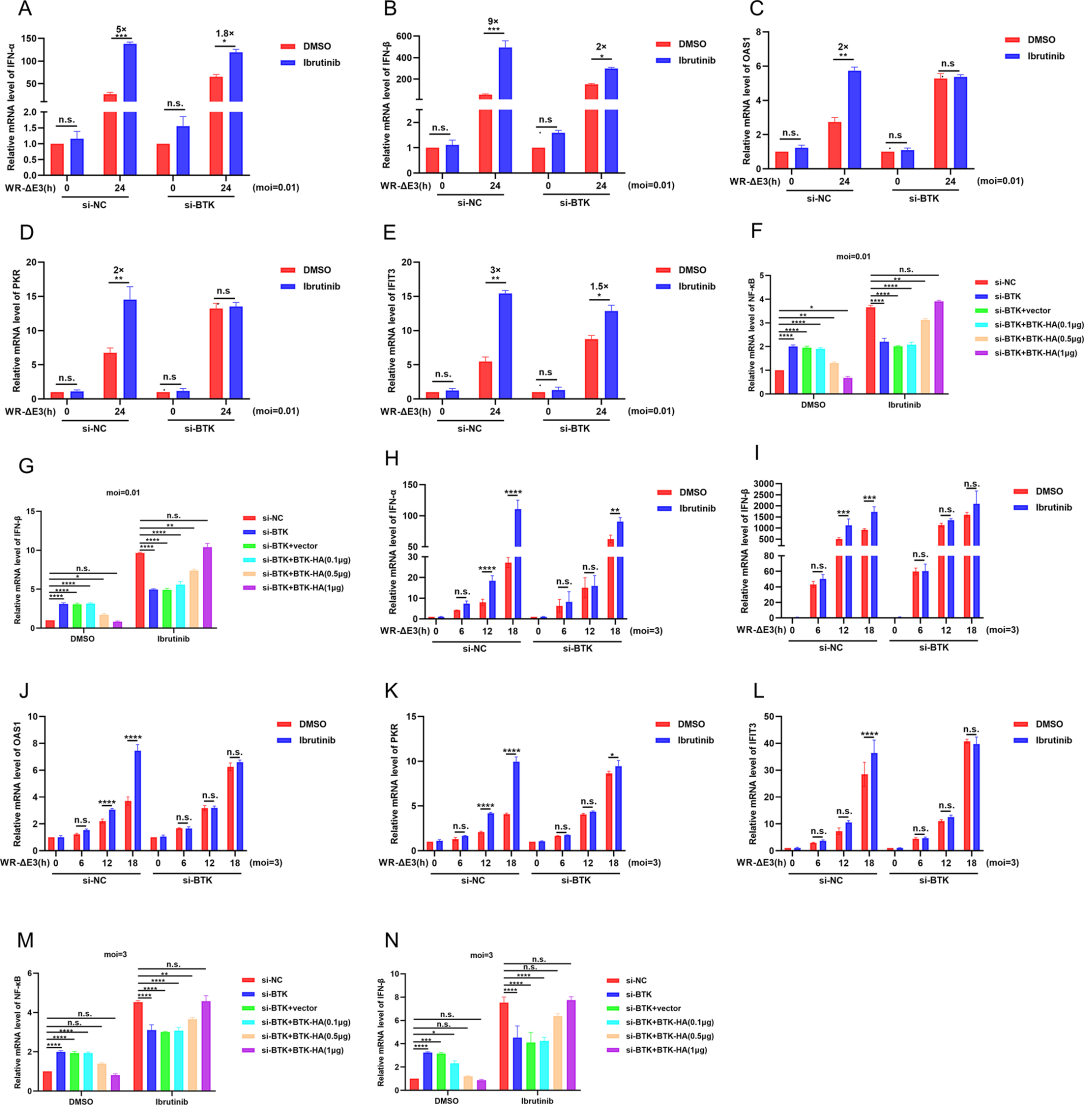
Ibrutinib promotes type I IFN response in a BTK-dependent manner. (**A–E**) HFF cells transfected with siNC (negative control) or siBTK were infected with WR-ΔE3 at 0.01 pfu/cell for 0 h or 24 h in the presence or absence of ibrutinib (10 µM). The relative mRNA levels of IFN-α, IFN-β, and the ISGs OAS1, PKR, and IFIT3 were examined by qRT-PCR. (**F and G**) HFF cells were transfected with siNC, siBTK, or BTK-HA (0.1, 0.5, 1 µg) for 24 h, and then cells were infected with WR-ΔE3 at 0.01 pfu/cell for 24 h in the presence or absence of ibrutinib (10 µM). The relative mRNA levels of NF-κB and IFN-β were examined by qRT-PCR. (**H–L**) HFF cells transfected with siNC (negative control) or siBTK were infected with WR-ΔE3 at 3 pfu/cell for 0, 6, 12, and 18 h in the presence or absence of ibrutinib (10 µM). The relative mRNA levels of IFN-α, IFN-β, and the ISGs OAS1, PKR, and IFIT3 were examined by qRT-PCR. (**M and N**) HFF cells were transfected with siNC, siBTK, or BTK-HA (0.1, 0.5, 1 µg) for 24 h, and then cells were infected with WR-ΔE3 at 3 pfu/cell for 18 h in the presence or absence of ibrutinib (10 µM). The relative mRNA levels of NF-κB and IFN-β were examined by qRT-PCR. Data in A–N are representative of three independent experiments. Statistics: n.s., not significant; *P* > 0.05; *P < 0.05; **P < 0.01; ***P < 0.001; ****P < 0.0001 by two-sided Student’s t-test.

BTK is primarily recognized as a positive regulator of NF-κB, especially in B cells (18, 32). However, emerging evidence demonstrated that BTK could also play a negative role in regulating NF-κB activation (33). We next asked whether ibrutinib impacts the induction of NF-κB and/or type I IFN responses upon VACV infection. HFF cells transfected with siNC (negative control) or siBTK for 24 h were treated with DMSO or ibrutinib (10 µM) and simultaneously infected with WR-ΔE3 (the E3L in VACV-WR was replaced with a cassette encoding eGFP, generating a recombinant virus named WR-ΔE3) at 0.01 pfu/cell for 0 or 24 h. Reverse transcription-quantitative PCR (RT-qPCR) analysis was then performed to assess the mRNA levels of IFNα, IFNβ, and selected ISGs, including OAS1, PKR, and IFIT3. As shown in Fig. 8A and B, infection with WR-ΔE3 increased the mRNA levels of IFNα and IFNβ. Importantly, ibrutinib treatment further enhanced the mRNA levels of both cytokines. In addition, the mRNA levels of selected ISGs (OAS1, PKR, and IFIT3) also exhibited an evident increase in response to ibrutinib treatment (Fig. 8C through E). Nevertheless, in cells transfected with siBTK, although the treatment of ibrutinib also led to an increase of type I IFNs, the level of induction was much less prominent than that in siNC-transfected cells (Fig. 8A and B). Similarly, the subsequent induction of ISGs was much less evident in siBTK-transfected cells than in siNC-transfected cells (Fig. 8C through E). Remarkably, in the absence of ibrutinib during viral infection, suppression of BTK by siRNA significantly induced NF-κB expression, and supplementation of BTK reversed this phenomenon in a dose-dependent manner (Fig. 8F), and similar results were observed for IFNβ (Fig. 8G). In order to further solidify our conclusion, HFF cells were infected with WR-ΔE3 at 3 pfu/cell for 0, 6, 12, and 18 h in the presence or absence of ibrutinib (10 µM). The relative mRNA levels of IFN-α, IFN-β, and the ISGs OAS1, PKR, and IFIT3 were examined by qRT-PCR. Infection with WR-ΔE3 increased the mRNA levels of IFNα, IFNβ, and the ISGs OAS1, PKR, and IFIT3 as the infection duration increased. Importantly, ibrutinib treatment further enhanced the mRNA levels of both cytokines and ISGs, including OAS1, PKR, and IFIT3 (Fig. 8H through L). Nevertheless, in cells transfected with siBTK, although the treatment of ibrutinib also led to an increase of type I IFNs, the level of induction was much less prominent than that in siNC-transfected cells (Fig. 8H and I). Similarly, the subsequent induction of ISGs was much less evident in siBTK-transfected cells than in siNC-transfected cells (Fig. 8J through L). In the absence of ibrutinib during viral infection, suppression of BTK by siRNA significantly induced NF-κB expression, and supplementation of BTK reversed this phenomenon in a dose-dependent manner (Fig. 8M), and similar results were observed for IFNβ (Fig. 8N). In summary, ibrutinib induced IFN-I responses in a BTK-dependent manner to exert antiviral effects. These results demonstrated that one of the mechanisms by which BTK promoted poxvirus replication was through dampening type I IFN responses.

## DISCUSSION

At present, mpox has endangered public health safety in our country, and the newly emerged bovine nodular skin disease has also caused great losses to the cattle breeding industry. Currently, there are no drugs with strong therapeutic efficacy against MPXV and LSDV, highlighting an urgent need for the development of new broad-spectrum anti-poxvirus treatments. In this context, targeting the interactions between poxviruses and host cell components is emerging as a promising strategy for antiviral therapy, as it is less likely to promote the emergence of drug-resistant viruses. Notably, some of these host cell processes are already targeted by existing drugs used to treat other diseases, paving the way for drug repurposing—the reapplication of known drugs for new therapeutic purposes. This approach can significantly accelerate the development of antiviral agents against poxviruses. In the present study, we explored the potential of ibrutinib in treating multiple poxvirus infections. As anticipated with a well-established and widely used drug, no toxicity was observed. More importantly, the compounds demonstrated high efficacy in limiting multiple poxvirus infections, underscoring their potential as effective antiviral therapies.

Tecovirimat (also known as TPOXX or ST-246) is a small-molecule intracellular viral release inhibitor, administered either orally or via intravenous injection. It has demonstrated specific efficacy against monkeypox virus, ectromelia virus, vaccinia virus, and other orthopoxviruses (34). In 2022, the European Medicines Agency (EMA) approved tecovirimat for the treatment of mpox; however, its widespread use remains limited (35). The drug is contraindicated in patients with severe renal insufficiency, and its common side effects include headache, nausea, abdominal pain, and vomiting (34, 36–38). Tecovirimat works by inhibiting the synthesis of the VARV VP37 protein and its homologs in other orthopoxviruses (39), a protein critical for the formation and release of enveloped viruses, which is associated with heightened virulence (40, 41). However, a single point mutation in the target protein may lead to antiviral resistance (42). In our study, we demonstrated that ibrutinib effectively reduces viral DNA levels, and suppression of its cellular target, BTK, also reduces poxvirus replication. Interestingly, we observed that BTK knockdown alone was less effective in blocking viral replication compared to treatment with ibrutinib. This suggests that ibrutinib may exert its antiviral effects through additional cellular mechanisms beyond BTK inhibition. Despite this, our findings confirm that BTK plays a pro-viral role in the replication of both VACV and MPXV in mammalian cells. Genetic suppression of BTK significantly inhibited viral replication, while ectopic expression of BTK promoted viral protein synthesis and replication.

In our study, treatment with the BTK inhibitor ibrutinib dramatically reduced pathogenic lesions in the lungs of infected mice relative to controls. Furthermore, ibrutinib delayed virus-induced weight loss and prolonged the survival time. Similar to our conclusion, Jon M. Florence showed that treatment with the BTK inhibitor not only reduced weight loss and death but also had a dramatic effect on morphological changes to the lungs in IAV-infected mice (19). Attenuation of lung inflammation indicative of acute lung injury, such as alveolar hemorrhage, interstitial thickening, and the presence of alveolar exudate, together with reduced levels of the inflammatory mediators TNFα, IL-1β, IL-6, KC, and MCP-1, strongly suggests amelioration of the pathological immune response in the lungs to promote resolution of the infection.

A previous study suggested that BTK suppression in HIV-1-infected cells using siRNA resulted in selective death of infected, but not uninfected cells (20). Using BTK-specific antibody and small-molecule inhibitors including LFM-A13 and an FDA-approved compound, ibrutinib (PCI-32765), Irene Guendel et al. have found that HIV-1-infected cells are sensitive to apoptotic cell death, resulting in a decrease in virus production. Overall, the data suggest that HIV-1-infected cells are more sensitive to BTK-targeted therapy in virus-infected cells (20). In our study, we found that genetic manipulation of BTK affected the replication of both VACV and MPXV, further demonstrating the involvement of BTK as an immune regulator during virus infection.

Once BTK is phosphorylated at Y551, it then autophosphorylates at Y223, which then triggers its full activation. In addition, C481 is the site to which BTK inhibitors covalently bind (30). In our investigation, the three key sites of BTK were also essential for the promotion of viral replication. Astoundingly, VACV infection led to the nuclear translocation of BTK, and the Y223 residue was essential for this process. These data suggested that the full activation of BTK is essential for its proviral function, and the process of which requires nuclear translocation of the kinase.

Yingying Li found that Syk is responsible for STAT1 activation at the early stage of IAV infection, which is critical for initial antiviral responses. Surprisingly, a time course study showed that Syk suppressed innate immunity during late phases of IAV infection and thereby promoted IAV replication. Disruption of Syk expression or blockade of Syk activation led to a significant increase in expression of type I IFNs (IFN-α and IFN-β) upon IAV infection in A549 and THP-1 cells (43). Syk deficiency enhanced the expression of type I and III interferons, inhibited IAV replication, and rendered mice more resistant to IAV infection. Syk impaired innate immune signaling through impeding TBK1 activation. These data reveal that Syk participates in the initiation of antiviral defense against IAV infection and simultaneously contributes to the restriction of innate immunity at the late stage of viral infection, suggesting that Syk serves a dual function in regulating antiviral responses. As BTK is a homologous protein of Syk, we hypothesized that BTK may facilitate viral infection by suppressing type I IFN responses in infected cells. As expected, pharmaceutical blockade of BTK activation by ibrutinib led to activation of the interferon response and subsequent expression of ISGs. Meanwhile, in BTK-KO cells, although ibrutinib treatment led to a marginal increase in the levels of type I interferon, this disparity was not significant when compared with wild-type (WT) cells. Even in BTK-KO cells treated with ibrutinib, there was no significant difference in ISGs (OAS1 and PKR) expression. In summary, ibrutinib enhances the type I interferon response in a BTK-dependent manner.

BTK exhibits a dual role in modulating NF-κB activation, acting as both an activator and inhibitor depending on the cellular context and external stimuli. Traditionally, BTK is recognized for its role in activating the NF-κB pathway, particularly in B cells, where it transmits signals from the B-cell receptor to promote NF-κB-mediated transcription essential for B-cell development and function (18). However, emerging evidence indicates that BTK can also function as a negative regulator of NF-κB activation in certain scenarios. For instance, studies have shown that cells lacking BTK exhibit increased NF-κB activation compared to wild-type cells (33), suggesting an inhibitory role for BTK in specific contexts. The dualistic nature of BTK’s regulation of NF-κB underscores the complexity of intracellular signaling networks and highlights the importance of context-dependent modulation in immune responses and viral pathogenesis.

## MATERIALS AND METHODS

### Drugs, plasmids, antibodies, cells, viruses, and animals

Ibrutinib (S2680) was purchased from Selleck.

pMLS-SV40-EGFP was purchased from addgene (#46919). BTK and its mutants (Y223F, C481Y, Y551F) plasmids were constructed by our lab.

Phospho-Btk (Tyr223) Antibody (#5082) was purchased from Cell Signaling Technology; BTK Antibody (CSB-PA002867LA01HU) was purchased from CUSABIO; Rabbit antibody to VACV-WR strain and mouse antibodies to VACV-I3 or VACV-E3 were described previously (44, 45); mouse anti-A3 and LSDV antibodies were prepared by our lab; mouse anti-GAPDH antibody (#AF0006), mouse anti-histone H3 antibody (#AF0009), mouse anti-HA antibody (#AH158), HRP-labeled goat anti-mouse IgG (#A0216), and HRP-labeled goat anti-rabbit IgG (#A0208) were purchased from Beyotime Biotechnology; Goat anti-rabbit IgG Alexa Fluor 555 (#A32732) was purchased from Thermo Fisher Scientific.

Alveolar type II-like epithelial (A549) cells and HeLa cells were purchased from Ningbo Ming Zhou Bio Co., Ltd. DF-1 (cat#CL-0279), MDBK[NBL-1] (cat#CL-0153) BHK-21[C-13] (cat#CL-0034) were kindly provided by Procell Life Science & Technology Co., Ltd. THP-1 cells were presented by the Academy of Military Medical Sciences. BS-C-1 and Vero cells were stored in our lab. All cells were maintained in Dulbecco’s modified Eagle’s medium (Solarbio, China) supplemented with 10% fetal bovine serum (BI Fetal Bovine Serum, US origin, 04-001-1ACS), 100 U/mL penicillin, and 100 μg/mL streptomycin at 37°C under 5% CO_2_.

VACV-WR and MVA were kindly provided by Professor Bernard Moss. LSDV-eGFP was constructed by Shijie Xie and reported previously (46). In this study, the mpox virus (GenBank: PP778666) was isolated from mpox patients. All mpox experiments were conducted in a Biosafety Level 3 laboratory, adhering strictly to standard operating procedures. A strain of the Vesicular stomatitis virus that contains an enhanced green fluorescent protein gene (VSV-eGFP) was kindly provided by Prof. ZhiGao Bu from the Harbin Veterinary Research Institute, China.

BS-C-1 cells were infected with VACV-WR at 0.05 pfu/cell and incubated for 2 days in a humidified 37°C 5% CO_2_ incubator. Detach cells by banging and scraping. Pipette up and down to resuspend and transfer to a 50 mL centrifuge tube. Spin 2,500 rpm, 4°C, 5–10 min to pellet cells and discard supernatant. Resuspend the pellet in 1.5 mL 10 mM Tris-Cl per T175 flask by pipetting. Keep samples on ice and lyse the cell suspension by homogenizing with 30–40 strokes in a glass dounce homogenizer with a tight pestle. Transfer to a conical tube and centrifuge 5 min at 1,000 rpm to remove nuclei. Save the supernatant and sonicate supernatants, keeping them on ice the entire time at 30%–40% power for 60 s. Repeat 3–4 times with 30 s rests in between on ice. Layer the sonicated lysate onto a cushion of 36% sucrose in a sterile centrifuge tube. Centrifuge 80 min at 32,900 *× g*. Aspirate and discard supernatant.

Vero E6 cells were infected with MPXV at 0.01 pfu/cell and incubated for 3 days in a humidified 37°C 5% CO_2_ incubator. Detach cells by banging and scraping. Collect cells and supernatant in 50 mL centrifuge tubes. Lyse the cell suspension by performing three freeze-thaw cycles, each time by freezing at −80°C, thawing at room temperature, and vortexing. Store the cell lysate at −80°C until needed in the experiments. All experiments with infectious MPXV were conducted in a biosafety level 3 laboratory.

Specific-pathogen-free BALB/c mice were purchased from Beijing Vital River Laboratory Animal Technology Co., Ltd. Seven- to eight-week-old female BALB/c mice were divided into four groups, and six female BALB/c mice were included in each group.

### Cell viability assays

A 3-(4,5-dimethylthiazol-2-yl)-2,5-diphenyl tetrazolium bromide (MTT) assay was performed using an MTT assay kit (Solarbio, M1020) to measure the cytotoxicity of the chemical inhibitors on cells. Briefly, A549, HeLa, DF1, and THP-1 cells in 96-well plates were cultured for 24 h at 37°C. After that, they were treated with DMSO or chemical inhibitors dissolved in DMSO and incubated for 24 h at 37°C. After incubation, 10 µL of MTT reagent was added to each well, and cells were incubated for another 4 h. Next, a 110 µL Formazan resolving solution was added to each well, and absorbance at 490 nm (UV light) was measured for each well.

### Viral infection and titration

A cell culture medium from cells at a 90% confluency was removed and replaced with MPXV, VACV-WR, MVA, LSDV-eGFP, HSV-1, and VSV-eGFP diluted in DMEM supplemented with 2.5% FBS. Cells were infected for 1 h at 37°C. The inoculum was then discarded, and cells were washed 2× times with PBS and maintained in a fresh medium. After 24 or 48 h, viruses from each treatment were collected by freezing/thawing three times, MPXV, VACV-WR, and HSV-1 virus titers were determined by plaque assay in BS-C-1 and Vero cells with viruses serially diluted in DMEM–2.5% FBS. MVA (47) or LSDV virus titers were determined by calculating stained foci using VACV antibody or LSDV074 (H3L) antibody immunostaining. VSV virus titers were determined by TCID50.

### Time-of-ibrutinib-addition assay

Time-of-addition assay was performed. Briefly, HeLa cells were incubated in a six-well plate overnight, and they were then inoculated with WR between 0 and 2 h. Ibrutinib (10 μM) or DMSO was used at five different time intervals. Viral titers were determined using plaque assays.

### *In vivo* anti-VACV-WR activity of ibrutinib

Seven- to eight-week-old female BALB/c mice were divided into four groups, six female BALB/c mice were included in each group, and 2 × 10^4^ pfu VACV-WR were intranasally inoculated into each mouse. Subsequently, ibrutinib suspended in 5% DMSO + 40% PEG300 + 5% Tween80 + 50% ddH_2_O was intranasally administered to mice at selected concentrations every day. Body weight and survival of the infected mice were monitored following infection. At 5 days post-infection, the viral yield in different organs was quantified via plaque assay. H&E staining and immunohistochemistry assay of sectioned lungs. At 2 days post-infection, pathological changes were evaluated.

### BTK knockdown and overexpression

HeLa cells were transfected with BTK-siRNA (siRNA-1#:5′-GCACAAACUCUCCUACUAUGATT-3′; siRNA-2#:5′-GACUCAUAUCCAGGCUCAAAUTT-3′; siRNA-3#:5′-GUAUAGCAAGUUCAGCAGCAATT-3′) for 48 h and were infected with WR at 0.01 pfu/cell. After 48 h, cells were lysed in SDS lysis buffer (Beyotime, China). A western blot was performed to detect the expression level of viral protein.

BTK was overexpressed in HeLa cells for 24 h, and HeLa cells were infected with 0.01 pfu/cell WR. After 48 h, a western blot was performed to detect the expression level of viral protein.

HeLa cells were infected with WR at 3 pfu/cell for 1, 3, 6, 9, and 12 h in the presence or absence of ibrutinib, and a western blot was performed to detect the expression level of phosphorylated BTK and total BTK protein. The ratio of phosphorylated BTK to total BTK protein level was measured and calculated by ImageJ software.

BTK mutants were overexpressed in HeLa-BTK-KO cells for 24 h, and HeLa cells were infected with 3 pfu/cell WR. After 24 h, a western blot was performed to detect the expression level of viral protein.

### Western blotting analysis

Cells were washed once with ice-cold PBS and lysed in wells with 1× cell lysis buffer and 1× PMSF (Beyotime Biotechnology) for western blotting analysis on ice. Proteins were resolved on 12% FastPAGE precast gels (Tsingke Biotechnology) and transferred to nitrocellulose membranes in a Trans-Blot Turbo machine (Bio-Rad). Membranes were blocked with 5% nonfat milk dissolved in Tris-buffered saline (TBS) for 1 h at RT and then incubated with primary antibodies diluted in 1× TBS with 0.1% Tween 20 (TBST) containing 5% nonfat milk overnight at 4°C. The membranes were washed three times with 1× TBST and incubated with a secondary antibody conjugated with horseradish peroxidase diluted 1:5,000 in 1× TBST containing 5% nonfat milk for 1 h at RT. ECL signals were detected with SuperSignal West Dura substrates (Thermo Fisher Scientific) and visualized by Image J.

### Quantification of viral genomic DNA and viral mRNA by qPCR

HeLa cells were infected with WR at 3 pfu/cell. Cells were washed twice with PBS and harvested for DNA or RNA extraction. Total DNA was isolated with QIAamp DNA Blood mini kit (Qiagen, 51104) at 8 hpi and quantitated by nanodrop spectrophotometer (ThermoFisher). Total RNA was extracted at 2 or 8 h after infection with RNAprep pure Cell/Bacteria kit (TIANGEN, DP430), quantitated with the nanodrop spectrophotometer, and equal amounts of RNA were treated with DNase prior to reverse-transcription with a First-Strand cDNA Synthesis SuperMix (TransGen, AE301-03). Equal amounts of DNA or cDNA from each sample were analyzed with gene-specific primers (E11L for viral genomic DNA) by qPCR according to the protocol described using TOROGreen qPCR Master Mix. Primers used for mRNA quantification:

qE3L-F: TGACAGGGTTAGCACCTTTCCAATC

qE3L-R: CGGATGCTGATGCTATGGCTGAC

qD13L-F: GATAGCCTGATTGTCTGGACCATCG

qD13L-R: AACGGTCTAATGTCTTCGCAGTCG

qA3L-F: CTATAGACAAAATAGAAGCC

qA3L-R: CCATGATTAGAAAAGCAATTATG

### Confocal microscopy

Monolayers of HeLa cells were grown on glass coverslips in 24-well plates and then infected with viruses indicated in Fig. 7A, B, and E for the indicated time. Medium was removed and cells were fixed with ice-cold 4% paraformaldehyde for 20 min at room temperature, followed by incubation with 0.1% Triton X-100 for 20 min and blocked with 3% BSA diluted in 1× PBS for 30 min. BTK antibody (1:200) was added and incubated with cells at 4°C overnight. Cells were then washed with 3% BSA three times, followed by incubation with secondary antibodies conjugated with Alexa Fluor 555 at 37°C in the dark with agitation for 1 h. Hoechst was used to stain the nucleus for 5 min. Cells were washed multiple times before mounting on slides using ProLong Diamond Antifade reagent (Thermo Fisher Scientific). Images were taken on a Leica SP8 confocal microscope, and images were processed with the Leica software (Leica Biosystems).

### Nucleocytoplasmic separation experiment

After HeLa cells were infected according to the specified time, the cytoplasmic and cytoplasmic protein extraction kit (P0027, Beyotime) was used to extract cytoplasmic and cytoplasmic protein, and BTK levels were detected by western blotting.

### Statistical analysis

Error bars represent the standard deviation. Asterisks denote the level of statistical significance, *P < 0.05, **P < 0.01, and ***P < 0.001, ****P < 0.0001, and n.s.: non-significant. Statistically significant differences between the two groups were estimated using an unpaired Student’s t-test. Multi-group comparison was performed using one-way analysis of variance (ANOVA) (Fig. 4D and 5). Survival curves were analyzed using the log-rank test. Statistical analyses were conducted using Prism 7 (GraphPad Software).

## Data Availability

All data generated and analyzed that support the findings of this study are included in the published article.

## References

[1] Thèves C, Crubézy E, Biagini P. 2016. History of smallpox and its spread in human populations. Microbiol Spectr 4. doi:10.1128/microbiolspec.PoH-0004-2014 27726788

[2] Dsouza L, Pant A, Offei S, Priyamvada L, Pope B, Satheshkumar PS, Wang Z, Yang Z. 2023. Antiviral activities of two nucleos(t)ide analogs against vaccinia, mpox, and cowpox viruses in primary human fibroblasts. Antiviral Res 216:105651. doi:10.1016/j.antiviral.2023.105651 37270160 PMC10234405

[3] Henderson DA. 2011. The eradication of smallpox--an overview of the past, present, and future. Vaccine (Auckl) 29 Suppl 4:D7–9. doi:10.1016/j.vaccine.2011.06.080 22188929

[4] Yang Z, Gray M, Winter L. 2021. Why do poxviruses still matter? Cell Biosci 11:96. doi:10.1186/s13578-021-00610-8 34022954 PMC8140567

[5] Woods JA. 1988. Lumpy skin disease--a review. Trop Anim Health Prod 20:11–17. doi:10.1007/BF02239636 3281337

[6] Namazi F, Khodakaram Tafti A. 2021. Lumpy skin disease, an emerging transboundary viral disease: a review. Vet Med Sci 7:888–896. doi:10.1002/vms3.434 33522708 PMC8136940

[7] Abutarbush SM, Ababneh MM, Al Zoubi IG, Al Sheyab OM, Al Zoubi MG, Alekish MO, Al Gharabat RJ. 2015. Lumpy skin disease in Jordan: disease emergence, clinical signs, complications and preliminary-associated economic losses. Transbound Emerg Dis 62:549–554. doi:10.1111/tbed.12177 24148185

[8] Hunter P, Wallace D. 2001. Lumpy skin disease in southern Africa: a review of the disease and aspects of control. J S Afr Vet Assoc 72:68–71. doi:10.4102/jsava.v72i2.619 11513262

[9] Davies FG. 1991. Lumpy skin disease, an African capripox virus disease of cattle. Br Vet J 147:489–503. doi:10.1016/0007-1935(91)90019-J 1777792

[10] Alu A, Lei H, Han X, Wei Y, Wei X. 2022. BTK inhibitors in the treatment of hematological malignancies and inflammatory diseases: mechanisms and clinical studies. J Hematol Oncol 15:138. doi:10.1186/s13045-022-01353-w 36183125 PMC9526392

[11] Vassilev A, Ozer Z, Navara C, Mahajan S, Uckun FM. 1999. Bruton’s tyrosine kinase as an inhibitor of the Fas/CD95 death-inducing signaling complex. J Biol Chem 274:1646–1656. doi:10.1074/jbc.274.3.1646 9880544

[12] Hendriks RW, Yuvaraj S, Kil LP. 2014. Targeting Bruton’s tyrosine kinase in B cell malignancies. Nat Rev Cancer 14:219–232. doi:10.1038/nrc3702 24658273

[13] Wen T, Wang J, Shi Y, Qian H, Liu P. 2021. Inhibitors targeting Bruton’s tyrosine kinase in cancers: drug development advances. Leukemia 35:312–332. doi:10.1038/s41375-020-01072-6 33122850 PMC7862069

[14] Gustafsson Manuela O, Hussain A, Mohammad DK, Mohamed AJ, Nguyen V, Metalnikov P, Colwill K, Pawson T, Smith CIE, Nore BF. 2012. Regulation of nucleocytoplasmic shuttling of Bruton’s tyrosine kinase (Btk) through a novel SH3-dependent interaction with ankyrin repeat domain 54 (ANKRD54). Mol Cell Biol 32:2440–2453. doi:10.1128/MCB.06620-11 22527282 PMC3434478

[15] Gustafsson MO, Mohammad DK, Ylösmäki E, Choi H, Shrestha S, Wang Q, Nore BF, Saksela K, Smith CIE. 2017. ANKRD54 preferentially selects Bruton’s Tyrosine Kinase (BTK) from a Human Src-Homology 3 (SH3) domain library. PLoS One 12:e0174909. doi:10.1371/journal.pone.0174909 28369144 PMC5378395

[16] Liu X, Pichulik T, Wolz O-O, Dang T-M, Stutz A, Dillen C, Delmiro Garcia M, Kraus H, Dickhöfer S, Daiber E, et al.. 2017. Human NACHT, LRR, and PYD domain-containing protein 3 (NLRP3) inflammasome activity is regulated by and potentially targetable through Bruton tyrosine kinase. J Allergy Clin Immunol 140:1054–1067. doi:10.1016/j.jaci.2017.01.017 28216434

[17] Page TH, Urbaniak AM, Espirito Santo AI, Danks L, Smallie T, Williams LM, Horwood NJ. 2018. Bruton’s tyrosine kinase regulates TLR7/8-induced TNF transcription via nuclear factor-κB recruitment. Biochem Biophys Res Commun 499:260–266. doi:10.1016/j.bbrc.2018.03.140 29567473 PMC5887515

[18] Mohamed AJ, Yu L, Bäckesjö C-M, Vargas L, Faryal R, Aints A, Christensson B, Berglöf A, Vihinen M, Nore BF, Smith CIE. 2009. Bruton’s tyrosine kinase (Btk): function, regulation, and transformation with special emphasis on the PH domain. Immunol Rev 228:58–73. doi:10.1111/j.1600-065X.2008.00741.x 19290921

[19] Florence JM, Krupa A, Booshehri LM, Davis SA, Matthay MA, Kurdowska AK. 2018. Inhibiting Bruton’s tyrosine kinase rescues mice from lethal influenza-induced acute lung injury. Am J Physiol Lung Cell Mol Physiol 315:L52–L58. doi:10.1152/ajplung.00047.2018 29516781 PMC6087894

[20] Guendel I, Iordanskiy S, Sampey GC, Van Duyne R, Calvert V, Petricoin E, Saifuddin M, Kehn-Hall K, Kashanchi F. 2015. Role of Bruton’s tyrosine kinase inhibitors in HIV-1-infected cells. J Neurovirol 21:257–275. doi:10.1007/s13365-015-0323-5 25672887 PMC4433585

[21] Lee KG, Xu S, Kang ZH, Huo J, Huang M, Liu D, Takeuchi O, Akira S, Lam KP. 2012. Bruton’s tyrosine kinase phosphorylates Toll-like receptor 3 to initiate antiviral response. Proc Natl Acad Sci USA 109:5791–5796. doi:10.1073/pnas.1119238109 22454496 PMC3326448

[22] Roschewski M, Lionakis MS, Sharman JP, Roswarski J, Goy A, Monticelli MA, Roshon M, Wrzesinski SH, Desai JV, Zarakas MA, Collen J, Rose K, Hamdy A, Izumi R, Wright GW, Chung KK, Baselga J, Staudt LM, Wilson WH. 2020. Inhibition of Bruton tyrosine kinase in patients with severe COVID-19. Sci Immunol 5:eabd0110. doi:10.1126/sciimmunol.abd0110 32503877 PMC7274761

[23] Bayerdörffer E, Neubauer A, Rudolph B, Thiede C, Lehn N, Eidt S, Stolte M. 1995. Regression of primary gastric lymphoma of mucosa-associated lymphoid tissue type after cure of Helicobacter pylori infection. MALT lymphoma study group. Lancet 345:1591–1594. doi:10.1016/s0140-6736(95)90113-2 7783535

[24] Kaliamurthi S, Selvaraj G, Selvaraj C, Singh SK, Wei DQ, Peslherbe GH. 2021. Structure-based virtual screening reveals ibrutinib and zanubrutinib as potential repurposed drugs against COVID-19. Int J Mol Sci 22:7071. doi:10.3390/ijms22137071 34209188 PMC8267665

[25] Peng C, Zhou Y, Cao S, Pant A, Campos Guerrero ML, McDonald P, Roy A, Yang Z. 2020. Identification of vaccinia virus inhibitors and cellular functions necessary for efficient viral replication by screening bioactives and FDA-approved drugs. Vaccines (Basel) 8:401. doi:10.3390/vaccines8030401 32708182 PMC7564539

[26] Yang Z, Reynolds SE, Martens CA, Bruno DP, Porcella SF, Moss B. 2011. Expression profiling of the intermediate and late stages of poxvirus replication. J Virol 85:9899–9908. doi:10.1128/JVI.05446-11 21795349 PMC3196450

[27] Quenelle DC, Kern ER. 2010. Treatment of vaccinia and Cowpox virus infections in mice with CMX001 and ST-246. Viruses 2:2681–2695. doi:10.3390/v2122681 21994637 PMC3185598

[28] Treon SP, Tripsas CK, Meid K, Warren D, Varma G, Green R, Argyropoulos KV, Yang G, Cao Y, Xu L, et al.. 2015. Ibrutinib in previously treated Waldenström’s macroglobulinemia. N Engl J Med 372:1430–1440. doi:10.1056/NEJMoa1501548 25853747

[29] Wang JD, Chen XY, Ji KW, Tao F. 2016. Targeting Btk with ibrutinib inhibit gastric carcinoma cells growth. Am J Transl Res 8:3003–3012. 27508020 PMC4969436

[30] Messex JK, Liou GY. 2021. Targeting BTK signaling in the microenvironment of solid tumors as a feasible cancer therapy option. Cancers (Basel) 13:2198. doi:10.3390/cancers13092198 34063667 PMC8124209

[31] Althubiti M, Rada M, Samuel J, Escorsa JM, Najeeb H, Lee K-G, Lam K-P, Jones GDD, Barlev NA, Macip S. 2016. BTK modulates p53 activity to enhance apoptotic and senescent responses. Cancer Res 76:5405–5414. doi:10.1158/0008-5472.CAN-16-0690 27630139

[32] Horwood NJ, Page TH, McDaid JP, Palmer CD, Campbell J, Mahon T, Brennan FM, Webster D, Foxwell BMJ. 2006. Bruton’s tyrosine kinase is required for TLR2 and TLR4-induced TNF, but not IL-6, production. J Immunol 176:3635–3641. doi:10.4049/jimmunol.176.6.3635 16517732

[33] Huang W, Morales JL, Gazivoda VP, August A. 2016. Nonreceptor tyrosine kinases ITK and BTK negatively regulate mast cell proinflammatory responses to lipopolysaccharide. J Allergy Clin Immunol 137:1197–1205. doi:10.1016/j.jaci.2015.08.056 26581914 PMC5730405

[34] Grosenbach DW, Honeychurch K, Rose EA, Chinsangaram J, Frimm A, Maiti B, Lovejoy C, Meara I, Long P, Hruby DE. 2018. Oral tecovirimat for the treatment of smallpox. N Engl J Med 379:44–53. doi:10.1056/NEJMoa1705688 29972742 PMC6086581

[35] Khamees A, Awadi S, Al-Shami K, Alkhoun HA, Al-Eitan SF, Alsheikh AM, Saeed A, Al-Zoubi RM, Zoubi MSA. 2023. Human monkeypox virus in the shadow of the COVID-19 pandemic. J Infect Public Health 16:1149–1157. doi:10.1016/j.jiph.2023.05.013 37269693 PMC10182868

[36] Adler H, Gould S, Hine P, Snell LB, Wong W, Houlihan CF, Osborne JC, Rampling T, Beadsworth MB, Duncan CJ, Dunning J, Fletcher TE, Hunter ER, Jacobs M, Khoo SH, Newsholme W, Porter D, Porter RJ, Ratcliffe L, Schmid ML, Semple MG, Tunbridge AJ, Wingfield T, Price NM, NHS England High Consequence Infectious Diseases (Airborne) Network. 2022. Clinical features and management of human monkeypox: a retrospective observational study in the UK. Lancet Infect Dis 22:1153–1162. doi:10.1016/S1473-3099(22)00228-6 35623380 PMC9300470

[37] Russo AT, Grosenbach DW, Chinsangaram J, Honeychurch KM, Long PG, Lovejoy C, Maiti B, Meara I, Hruby DE. 2021. An overview of tecovirimat for smallpox treatment and expanded anti-orthopoxvirus applications. Expert Rev Anti Infect Ther 19:331–344. doi:10.1080/14787210.2020.1819791 32882158 PMC9491074

[38] Quenelle DC, Buller RML, Parker S, Keith KA, Hruby DE, Jordan R, Kern ER. 2007. Efficacy of delayed treatment with ST-246 given orally against systemic orthopoxvirus infections in mice. Antimicrob Agents Chemother 51:689–695. doi:10.1128/AAC.00879-06 17116683 PMC1797744

[39] Payne LG. 1980. Significance of extracellular enveloped virus in the in vitro and in vivo dissemination of vaccinia. J Gen Virol 50:89–100. doi:10.1099/0022-1317-50-1-89 7441216

[40] Smith GL, Vanderplasschen A, Law M. 2002. The formation and function of extracellular enveloped vaccinia virus. J Gen Virol 83:2915–2931. doi:10.1099/0022-1317-83-12-2915 12466468

[41] Berhanu A, King DS, Mosier S, Jordan R, Jones KF, Hruby DE, Grosenbach DW. 2010. Impact of ST-246(R) on ACAM2000 smallpox vaccine reactogenicity, immunogenicity, and protective efficacy in immunodeficient mice. Vaccine 29:289–303. doi:10.1016/j.vaccine.2010.10.039 21036130 PMC3023305

[42] Lantto J, Haahr Hansen M, Rasmussen SK, Steinaa L, Poulsen TR, Duggan J, Dennis M, Naylor I, Easterbrook L, Bregenholt S, Haurum J, Jensen A. 2011. Capturing the natural diversity of the human antibody response against vaccinia virus. J Virol 85:1820–1833. doi:10.1128/JVI.02127-10 21147924 PMC3028881

[43] Li Y, Liu S, Chen Y, Chen B, Xiao M, Yang B, Rai KR, Maarouf M, Guo G, Chen JL. 2022. Syk facilitates influenza A virus replication by restraining innate immunity at the late stage of viral infection. J Virol 96. doi:10.1128/jvi.00200-22 PMC9006912 35293768

[44] Davies DH, Wyatt LS, Newman FK, Earl PL, Chun S, Hernandez JE, Molina DM, Hirst S, Moss B, Frey SE, Felgner PL. 2008. Antibody profiling by proteome microarray reveals the immunogenicity of the attenuated smallpox vaccine modified vaccinia virus ankara is comparable to that of Dryvax. J Virol 82:652–663. doi:10.1128/JVI.01706-07 17977963 PMC2224576

[45] Lin Y-CJ, Li J, Irwin CR, Jenkins H, DeLange L, Evans DH. 2008. Vaccinia virus DNA ligase recruits cellular topoisomerase II to sites of viral replication and assembly. J Virol 82:5922–5932. doi:10.1128/JVI.02723-07 18417590 PMC2395158

[46] Xie S, Cui L, Liao Z, Zhu J, Ren S, Niu K, Li H, Jiang F, Wu J, Wang J, Wu J, Song B, Wu W, Peng C. 2024. Genomic analysis of lumpy skin disease virus asian variants and evaluation of its cellular tropism. NPJ Vaccines 9:65. doi:10.1038/s41541-024-00846-8 38514651 PMC10957905

[47] Navarro-Forero S, Dsouza L, Yang Z. 2025. Modified vaccinia virus ankara titration using crystal violet- or immuno-staining in DF-1 cells. Methods Mol Biol 2860:287–296. doi:10.1007/978-1-0716-4160-6_19 39621275

